# Successful Treatment of Occlusive Left Main Coronary Artery Dissection by Impella-Supported Stenting

**DOI:** 10.1155/2018/5373625

**Published:** 2018-07-15

**Authors:** James J. Glazier, Amir Kaki, Theodore L. Schreiber

**Affiliations:** Detroit Medical Center Heart Hospital, Wayne State University, Detroit, MI, USA

## Abstract

We report successful treatment of a patient, who, during diagnostic angiography, developed an ostial left main coronary artery dissection with stump occlusion of the vessel. First, mechanical circulatory support with an Impella CP device was established. Then, patency of the left coronary system was achieved by placement of stents in the left anterior descending, left circumflex, and left main coronary arteries. On completion of the procedure, left ventricular systolic function, as assessed by echocardiography, was normal. At 24-month clinical follow-up, the patient remains angina-free and well. This is the first reported case of the use of an Impella device to support treatment of iatrogenic left main coronary artery dissection.

## 1. Introduction

The Impella device is a percutaneously inserted miniaturized ventricular assist device that is being increasingly used in the treatment of patients with acute myocardial infarction complicated by cardiogenic shock (AMICS) [1–4]. It has also been found to have a potentially valuable role in increasing the safety and efficacy of high-risk coronary interventional procedures (PCI), such as stenting of unprotected left main stem coronary artery (ULMCA) [5]. Using a retrograde femoral (or, occasionally, axillary) artery access, it is generally placed using standard percutaneous techniques in the left ventricular chamber (LV) across the aortic valve. The device pumps blood from the left ventricle into the ascending aorta and systemic circulation at an upper rate between 2.5 and 5.0 L/min, depending on the particular model type. The device provides almost immediate and sustained unloading of the left ventricle while increasing overall systemic cardiac output with maintenance of mean arterial pressure [1, 2]. Recently, an increasing body of evidence has suggested that in AMICS, a strategy of first implanting the Impella device before performing PCI is associated with improved survival [3, 4]. In particular, a recent report by Meraj et al. [6] suggests that initiation of cardiac mechanical support by Impella prior to PCI on ULMCA culprit lesion in AMCIS is associated with significantly improved early survival compared to Impella support following PCI. We now report our application of these observations in the treatment of a patient with catheter-induced obliterative occlusion of the LMCA.

## 2. Case Report

A 58-year-old woman with a history of current cigarette smoking, hypertension, and hyperlipidemia presented to the emergency room at our center reporting recurrent episodes of severe central chest pain over the preceding 24 hours. While her ECG showed no significant ST segment shifts, troponin I levels were slightly increased (0.025 ng/mL). Accordingly, she was referred for coronary angiography in the setting of a non-ST segment elevation MI.

**Figure 1:**
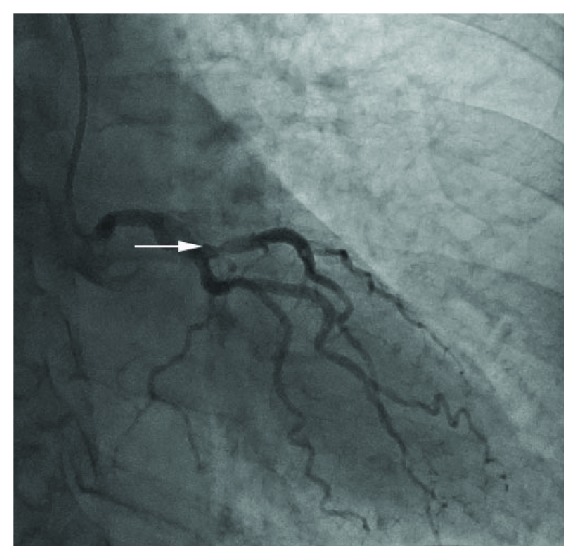
Left coronary angiogram showing a moderate stenosis (arrow) of the left anterior descending coronary artery.

**Figure 2:**
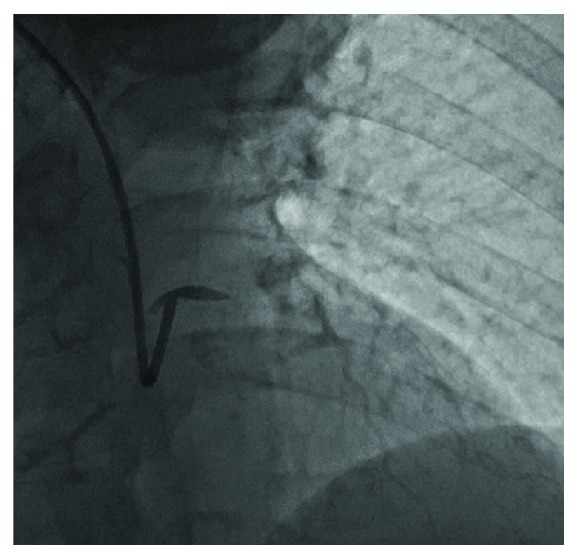
Occlusive dissection of the left main coronary artery.

Catheterization was performed via the right radial artery using the 6 French (F) Amplatz R1 and 6F Judkins L 3.5 diagnostic catheters (Medtronic Inc., Minneapolis, MN, USA). The only angiographic abnormality noted was a moderate stenosis of the mid left anterior descending coronary artery (LAD) (Figure 1). To further assess the physiological significance of this stenosis, an iFFR PrimeWire (Volcano Corp, San Diego, CA, USA) was placed in the LAD, following exchange of the Judkins catheter for a 6F Extra Back-Up (EBU) 3.5 guiding catheter (Medtronic Inc., Minneapolis, MN, USA). Of note, initial angiography through the EBU guide catheter prior to advancing the wire showed good coronary flow. It was, however, not possible to advance the wire to the lesion. Accordingly, the PrimeWire was removed from the vessel and then an angiogram of the left coronary artery was taken. Angiography revealed only a stump of the left main coronary artery (LMCA) with occlusion of both the LAD and of the circumflex (LCx) coronary arteries (Figure 2). Marked (3 mm) anterior ST segment elevation then developed, and the patient became progressively hypotensive with systolic pressure falling to a nadir of 58 mmHg. Inotropic and pressor infusions were commenced.

We, at this point, decided to establish mechanical circulatory support with the Impella CP device (Abiomed, Danvers, MA, USA) prior to attempting to reestablish patency of the left coronary system with stents. We first placed a 6F sheath in the left femoral artery. Just as we gained access, the patient developed ventricular fibrillation. This was immediately treated with one 150-Joule biphasic nonsynchronized shock. Following this, the 6F sheath was changed out over a 0.35^″^ Wholey wire (Medtronic Inc., Minneapolis, MN, USA) for a long 8F arterial sheath. Then, a 6F multipurpose diagnostic catheter was advanced through the sheath to the LV. Next, the Impella 0.18^″^ deployment wire was advanced through the multipurpose catheter to the LV and the multipurpose catheter then withdrawn. The 8F sheath was then changed out for a 14F sheath, and the Impella CP device advanced over the wire to the LV. The wire was withdrawn, and satisfactory positioning of the device was confirmed by fluoroscopy with the inflow within the LV and the outflow above the aortic valve. The Impella device was then activated and placed on “auto mode” which allows the device to ramp to P9 level resulting in a cardiac output of 3.5 L/min. The mean arterial pressure increased to 80 mmHg, and there was no further occurrence of ventricular dysrhythmias.

Having initiated mechanical circulatory support, we then set out to reestablish patency of the left coronary system. A 0.014^″^ Runthrough guidewire (Terumo, Somerset, NJ, USA) was advanced into the LCx artery, and a second 0.014^″^ Runthrough wire advanced into the LAD. The vessels were then dilated with 2.0 mm × 20 mm and 2.5 mm × 20 mm Trek (Abbott Vascular, Santa Clara, CA, USA) balloons with restoration of flow first in the LAD and then in the LCx.

**Figure 3:**
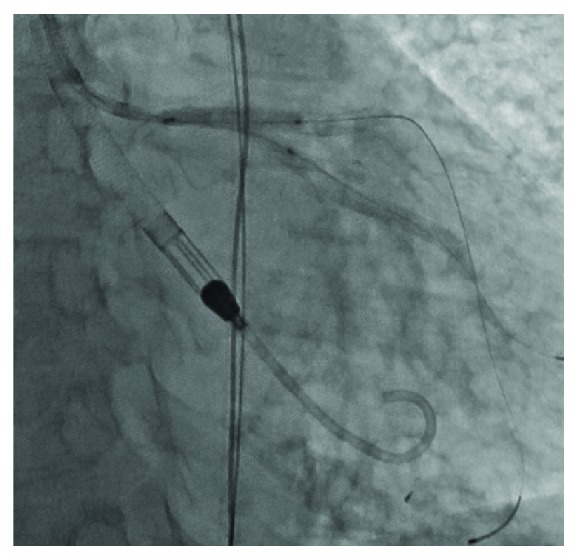
Final deployment of the left anterior descending and left circumflex coronary artery stents with a kiss.

**Figure 4:**
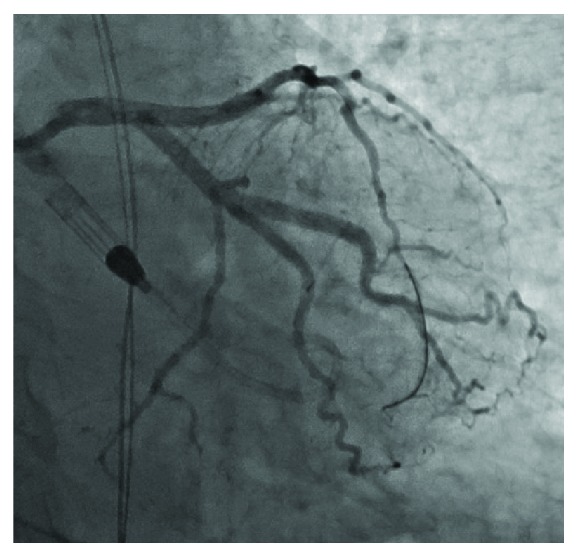
Final angiographic result demonstrating patency of the left coronary system.

**Figure 5:**
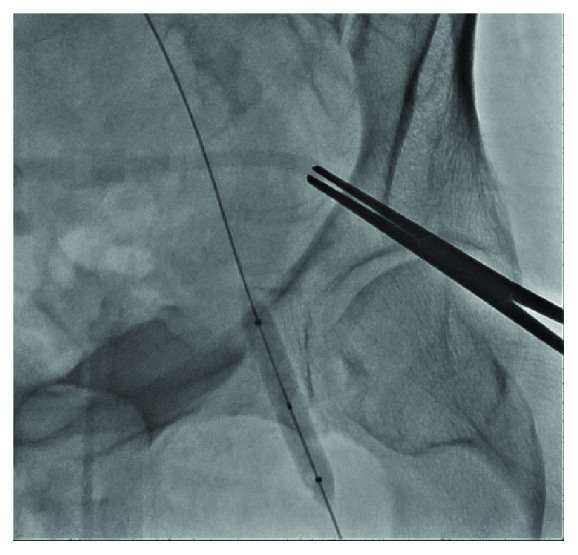
Crossover balloon tamponade of the left femoral artery following removal of the Impella device.

**Figure 6:**
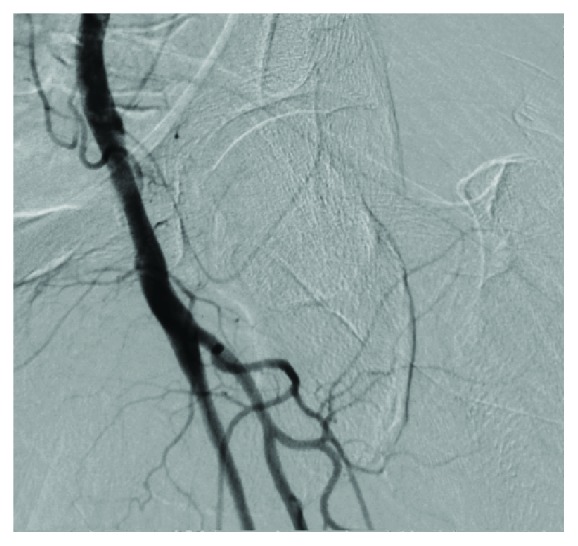
Final left femoral angiogram confirming hemostasis and absence of femoral artery abnormality or complication.

We then placed a 3.0 mm × 18 mm Resolute stent (Medtronic Inc., Minneapolis, MN, USA) from the LM to the LAD. Next, we rewired the LCx through the LAD stent struts. We then stented the LCx with a 3.0 mm × 38 mm Resolute stent. After this, a 3.0 mm × 20 mm Trek balloon was placed within the LAD stent, and a 2.5 mm × 15 mm Trek balloon placed within the LCx stent. Final deployment of the stents was with simultaneous inflation of the 2 balloons (kissing balloon dilation) (Figure 3). Finally, a 4.0 mm × 9 mm Resolute stent was deployed from the shaft to the ostium of the LM. Angiography demonstrated reestablishment of patency of the LM, LAD, and LCx (Figure 4), and intravascular ultrasound of the LAD and LM showed good stent apposition in these vessels. Immediately on completion of the procedure, a 2D echocardiogram was performed in the lab. This showed normal LV systolic function (LVEF = 55%). By this time, all inotropic and pressor agents had been discontinued. The patient was then weaned from the Impella device, and the device was removed from the cardiac catheterization laboratory. At our center, the usual approach to device removal is with suture-mediated closure. Because of the emergent need for Impella support in our patient, a technique of crossover balloon tamponade was used (Figure 5) with completion angiography to confirm hemostasis and absence of femoral artery abnormality or complication (Figure 6). The general approach at our center to cases of primary PCI in the setting of cardiogenic shock is to retain the Impella hemodynamic support for a minimum of 24 hours to allow for myocardial recovery. However, in this case, because of the rapid identification of shock and the prompt recovery of mean arterial pressure and the ability to discontinue all pressor support, we elected to remove the device while in the cardiac catheterization laboratory. An additional parameter that supported the latter decision was demonstration of a mixed venous oxygen saturation ≥ 65% on P3 setting for 30 minutes.

**Figure 7:**
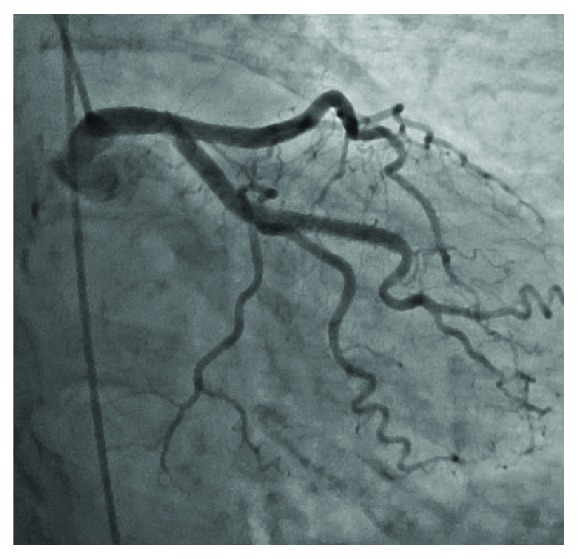
Left coronary angiogram at 6-month follow-up showing continued patency of the previously placed stents.

Following her procedure, the patient did well with no recurrence of symptoms, hemodynamic abnormalities, or dysrhythmias, and she was discharged home 2 days later. Follow-up coronary angiography 6 months after the initial procedure (Figure 7) showed continuing patency of the left coronary system without any significant residual stenosis. At 24-month clinical follow-up, the patient remains angina-free and with continuing normal LV systolic function.

## 3. Discussion

Iatrogenic LMCA dissection, although rare, is a dreaded complication of diagnostic coronary angiography, often dubbed “the angiographer's nightmare.” It has potentially devastating complications, including death on the cath lab table. It is usually treated with immediate stenting [7–10]. However, we judged that initial stenting might not be the optimal initial strategy in our patient. Most reports regarding iatrogenic LMCA dissection have described patients with a nonocclusive pattern with residual continued flow in the LAD and LCx. In contrast, our patient had an extreme form of dissection with amputation of the LMCA and no flow in the LAD or LCx. With such a dissection, successful advancement of guidewires first into the true lumen of the LMCA and then into the true lumina of the LAD and LCx can be technically challenging and time-consuming, with no guarantee of eventual success. Moreover, our patient was hemodynamically unstable and needed urgent institution of additional circulatory support to prevent development of refractory cardiogenic shock.

Of the available mechanical support devices, the Impella device seemed particularly suited to our patient. This device can be inserted quickly and provides almost immediate unloading of the LV and increased cardiac output and maintenance of mean arterial pressure. The particular Impella model used in our patient was the Impella CP. This pumps blood from the LV to the systemic circulation at a rate up to 4.0 L/min. This effect was demonstrated in our patient, who became hemodynamically stable within a few minutes of initiation of the Impella support. Thus, it allowed us to undertake, in a stable setting, the technically demanding and complex task of wiring and then performing bifurcation stenting using a two-stent technique of the occluded left coronary system. This stability was likely a key factor in achieving the excellent angiographic and durable clinical results out to 24 months seen in our patient. Without the Impella device, we would likely have had to attempt complex PCI on a background of recurrent malignant ventricular dysrhythmias and progressive hypotension despite escalating doses of inotropes and pressors. The Impella device also served as a potential valuable bridge to coronary bypass surgery in the event that we were unsuccessful in restoring left coronary patency by PCI.

Other authors [8, 10] have described the use of the intra-aortic balloon pump (IABP), as opposed to the Impella device, to provide hemodynamic support during coronary stent placement for left main dissection. However, in comparison to the Impella CP device, the ability of IABP to augment cardiac output is very modest: no more than 0.5 L/min [1]. The superior hemodynamic effect of the Impella device was a key factor in choosing this device over IABP in our patient. In addition, our large experience with the Impella (we implant >150 devices per year) allows us to implant this device in about the same time it takes to implant an IABP. However, we concede that there is a lack of evidence based on randomized controlled clinical trials to favor the use of the Impella over the IABP either in patients with cardiogenic shock [11] or in patients undergoing high-risk PCI [12].

Recently, an increasing body of evidence suggests that in patients with cardiogenic shock (as was the case for our patient), early initiation of Impella (i.e., initiation before PCI) (as was done in our patient) is associated with increased patient survival when compared with a strategy of initiating Impella after PCI [3, 4, 6]. In the reported data set, early initiation of Impella provides effective left ventricular unloading while maintaining adequate systemic and coronary perfusion and thus prevents the downward spiral of cardiogenic shock. These data provide further support for the interventional strategy used in the presently reported patient.

If the operator and cardiac catheterization laboratory have limited experience in Impella implantation, an excessive amount of time may be spent in attempting to implant the device. Accordingly, in such situations, it may be preferable that the operator proceeds directly to attempting to wire the occluded vessel.

The results of a recently reported study suggest that in non-CS patients undergoing LMCA PCI, prophylactic insertion of the Impella ventricular assist device may be associated with improved procedural success rates and reduced complication rates [5]. Accordingly, Impella insertion prior to LMCA stenting may also be considered in selected hemodynamically stable patients with iatrogenic LMCA dissection.

## 4. Conclusion

In the treatment of iatrogenic LMCA dissection, a strategy of initial insertion of the Impella device followed by LMCA stenting should be considered favorably in all those with hemodynamic instability as well as in selected hemodynamically stable patients.

## References

[1] Burzotta F., Trani C., Doshi S. N. (2015). Impella ventricular support in clinical practice: collaborative viewpoint from a European expert user group. *International Journal of Cardiology*.

[2] Gilotra N. A., Stevens G. R. (2015). Temporary mechanical circulatory support: a review of the options, indications, and outcomes. *Clinical Medicine Insights: Cardiology*.

[3] Basir M. B., Schreiber T. L., Grines C. L. (2017). Effect of early initiation of mechanical circulatory support on survival in cardiogenic shock. *The American Journal of Cardiology*.

[4] Flaherty M. P., Khan A. R., O’Neill W. W. (2017). Early initiation of Impella in acute myocardial infarction complicated by cardiogenic shock improves survival: a meta-analysis. *JACC: Cardiovascular Interventions*.

[5] Schreiber T., Wah Htun W., Blank N. (2017). Real-world supported unprotected left main percutaneous coronary intervention with Impella device; data from the USpella registry. *Catheterization and Cardiovascular Interventions*.

[6] Meraj P. M., Doshi R., Schreiber T., Maini B., O'Neill W. W. (2017). Impella 2.5 initiated prior to unprotected left main PCI in acute myocardial infarction complicated by cardiogenic shock improves early survival. *Journal of Interventional Cardiology*.

[7] Eshtehardi P., Adorjan P., Togni M. (2010). Iatrogenic left main coronary artery dissection: incidence, classification, management, and long-term follow-up. *American Heart Journal*.

[8] Kubota H., Nomura T., Hori Y. (2017). Successful bailout stenting strategy against lethal coronary dissection involving left main bifurcation. *Clinical Case Reports*.

[9] Akgul F., Batyraliev T., Besnili F., Karben Z. (2016). Emergency stenting of unprotected left main coronary artery after acute catheter-induced occlusive dissection. *Texas Heart Institute Journal*.

[10] Onsea K., Kayaert P., Desmet W., Dubois C. L. (2011). Iatrogenic left main coronary artery dissection. *Netherlands Heart Journal*.

[11] Glazier J. J., Kaki A. (2018). Improving survival in cardiogenic shock: is Impella the answer?. *The American Journal of Medicine*.

[12] O'Neill W. W., Kleiman N. S., Moses J. (2012). A prospective, randomized clinical trial of hemodynamic support with Impella 2.5 versus intra-aortic balloon pump in patients undergoing high-risk percutaneous coronary intervention: the PROTECT II study. *Circulation*.

